# Generation of human induced pluripotent stem cell-derived liver buds with chemically defined and animal origin-free media

**DOI:** 10.1038/s41598-020-73908-1

**Published:** 2020-10-21

**Authors:** Keisuke Sekine, Shimpei Ogawa, Syusaku Tsuzuki, Tatsuya Kobayashi, Kazuki Ikeda, Noriko Nakanishi, Kenta Takeuchi, Eriko Kanai, Yugo Otake, Satoshi Okamoto, Tsuyoshi Kobayashi, Takanori Takebe, Hideki Taniguchi

**Affiliations:** 1 Division of Regenerative Medicine, Center for Stem Cell Biology and Regenerative Medicine, The Institute of Medical Science, The University of Tokyo, Tokyo, 108-8639 Japan; 2 Department of Regenerative Medicine, Yokohama City University Graduate School of Medicine, Yokohama, 236-0004 Japan; 3 Research Institute for Bioscience Products and Fine Chemicals, Ajinomoto Co., Inc., Kawasaki, 210-8681 Japan; 4 Advanced Medical Research Center, Yokohama City University, Yokohama, 236-0004 Japan; 5 Division of Gastroenterology, Hepatology and Nutrition, Developmental Biology, Center for Stem Cell and Organoid Medicine (CuSTOM), Cincinnati Children’s Hospital Medical Center, Cincinnati, OH 45229-3039 USA; 6 Department of Pediatrics, University of Cincinnati College of Medicine, Cincinnati, OH 45229-3039 USA

**Keywords:** Composites, Electronic properties and materials, Nanowires, Structural properties

## Abstract

Advances in organoid technology have broadened the number of target diseases and conditions in which human induced pluripotent stem cell (iPSC)-based regenerative medicine can be applied; however, mass production of organoids and the development of chemically defined, animal origin-free (CD-AOF) media and supplements are unresolved issues that hamper the clinical applicability of these approaches. CD-AOF media and supplements ensure the quality and reproducibility of culture systems by lowering lot-to-lot variations and the risk of contamination with viruses or toxins. We previously generated liver organoids from iPSCs, namely iPSC-liver buds (iPSC-LBs), by mimicking the organogenic interactions among hepatocytes, endothelial cells (ECs), and mesenchymal cells (MCs) and recently reported the mass production of iPSC-LBs derived entirely from iPSCs (all iPSC-LBs), which should facilitate their large-scale production for the treatment of liver failure. However, in previous studies we used media originating from animals for differentiation except for the maintenance of undifferentiated iPSCs. Therefore, we developed a CD-AOF medium to generate all iPSC-LBs. We first developed a CD-AOF medium for hepatocytes, ECs, and stage-matched MCs, i.e., septum transversum mesenchyme (STM), in 2D cultures. We next generated all iPSC-LBs by incubating individual cell types in ultra-low attachment micro-dimple plates. The hepatic functions of all iPSC-LBs generated using the CD-AOF medium were equivalent to those of all iPSC-LBs generated using the conventional medium both in vitro and in vivo. Furthermore, we found that this CD-AOF medium could be used in several cell culture settings. Taken together, these results demonstrate the successful development of a CD-AOF medium suitable for all iPSC-LBs. The protocol developed in this study will facilitate the clinical applicability of all iPSC-LBs in the treatment of liver diseases.

## Introduction

Human induced pluripotent stem cells (iPSCs) hold great promise for regenerative medicine^1,2^, and in the last ten years iPSC-derived cells have been successfully transplanted into patients^3^. Important improvements have been achieved in recent years to ensure the safety and quality of iPSC culture systems for clinical application. Retrovirus and lentivirus used to transduce iPSCs into host cells in initial experiments have been replaced by methods that do not require integration into host chromosomes, thereby avoiding insertion mutations and potentially malignant transformations^4,5^. Undifferentiated iPSC culture conditions were also improved with the development of feeder-free (Ff) culture conditions and chemically defined, animal origin-free (CD-AOF) iPSC medium for use in clinical applications^6–10^.

We have recently succeeded in generating multicellular liver organoids from human iPSCs which we have defined as iPSC-liver buds (LBs); these LBs exhibit therapeutic potential in mouse disease models^11^. Single-cell RNA sequencing has revealed that the iPSC-LBs exhibit a clear resemblance to fetal liver tissues compared to the 2D-differentiated hepatocytes^12^. Furthermore, we have recently reported the mass production of iPSC-LBs derived entirely from human Ff-iPSCs, which we have termed all iPSC-LBs^13^. In addition to hepatic lineages, endothelial cells (ECs) and stage-matched mesenchymal cells (MCs), such as septum transversum mesenchymal cells (STM), have been derived from clinically applicable Ff-iPSCs. While these approaches will facilitate the large-scale production of iPSC-LBs for treating liver failure, which requires 1 × 10^8^–1 × 10^10^ hepatocyte-equivalents, the use of serum and animal-derived products must be reduced or completely eliminated during the production of all iPSC-LBs to meet the Standard for Biological Ingredients (The Japanese Ministry of Health, Labour and Welfare Notification No. 375 2014) during therapeutic applications.

In this study, we optimized the protocol for generating iPSC-LBs and developed a CD-AOF medium for clinical use. We examined the generation of hepatic, endothelial, and mesenchymal lineages from iPSCs using the CD-AOF medium. The resultant cells were combined to generate all iPSC-LBs, whose hepatic functions were equivalent to those of LBs generated using the conventional media both in vitro and in vivo. In addition, this CD-AOF medium, which we have defined as StemFit for differentiation (AS400), might be useful as a differentiation supplement for multiple lineage-derived cells.

## Results

### Efficient and clinical differentiation of human iPSCs to LBs

**Figure 1:**
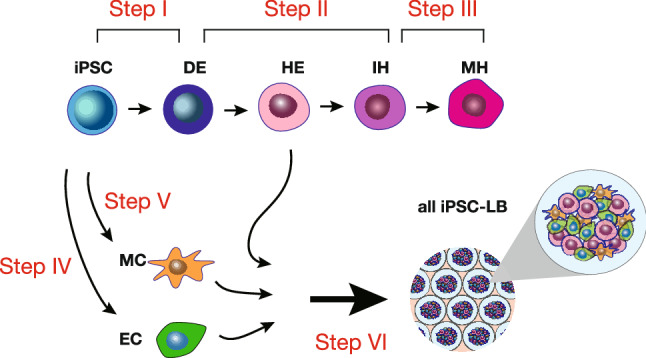
Schematic representation of the generation of liver buds and the media. The current study includes a total of six media formulations, including three formulations for differentiating hepatic lineages, one formulation each for differentiating mesenchymal and endothelial cells, and one formulation for generating liver buds (LBs) (steps I–VI).

A minimum of six different media are used in the conventional protocol to generate all iPSC-LBs (Fig. 1). We first developed three distinct media to differentiate iPSCs into mature hepatocytes (MHs) through definitive endoderm (DE), hepatic endoderm (HE), and immature hepatocyte (IH) stages in a 2D culture system (Fig. 1 and “Methods”). We used clinically applicable iPSC lines established using nonintegrating episomal vectors at Kyoto University to derive hepatic, endothelial, and mesenchymal lineages. These cells were generated under Ff conditions using the CD-AOF media and maintained under identical conditions (see “Methods”)^6–10^.

**Figure 2:**
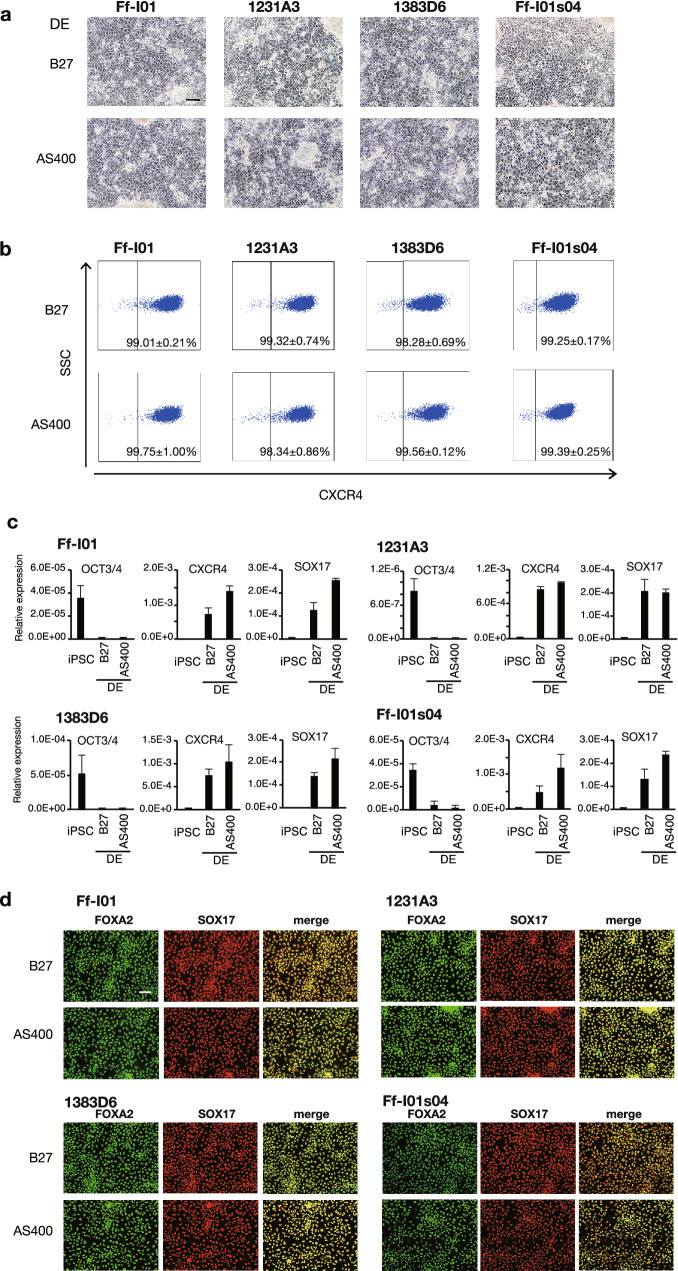
Generation of definitive endoderm using defined medium (Step I). (**a**) Morphological representation of definitive endoderm (DE) differentiated from four different induced pluripotent stem cell (iPSC) lines using conventional or chemically defined, animal origin-free (CD-AOF) medium. Scale bar, 100 µm. (**b**) Flow cytometric analysis of the CXCR4 expression in DEs differentiated using the conventional or the CD-AOF medium. (**c**) Gene expression analysis of the DEs differentiated using the conventional or the CD-AOF medium. Error bars represent standard deviation. (**d**) Immunostaining of the DE for FOXA2 and SOX17. Scale bar, 100 µm.

We and others previously reported the use of B27™ supplement for serum replacement during the first step of DE differentiation^14–16^. We examined the possibility of replacing B27, Activin A, and Wnt-3a in the conventional DE differentiation medium with the CD-AOF supplement AS400, human recombinant activin A, and the glycogen synthase kinase 3 inhibitor CHIR99021, respectively. At day six, the cells appeared normal, and their morphology was similar to that of cells differentiated using the conventional medium (Fig. 2a). The resultant DE was evaluated using flow cytometric and gene expression analyses. After the DE induction, the percentage of C-X-C chemokine receptor type 4 (CXCR4)-positive cells in multiple iPSC clones ranged from 98.28 to 99.32% in the conventional medium and from 98.34 to 99.75% in the CD-AOF medium (Fig. 2b). The expression levels of several DE markers including SRY-box transcription factor 17 (SOX17) and CXCR4 were comparable between the DEs generated using the conventional and CD-AOF media (Fig. 2c). The immunohistochemical analyses revealed similar findings, as expected from the gene expression and flow cytometric analyses (Fig. 2d). These results indicate that the DE was successfully differentiated using the CD-AOF medium with efficiency that was comparable to that of the conventional medium.

**Figure 3:**
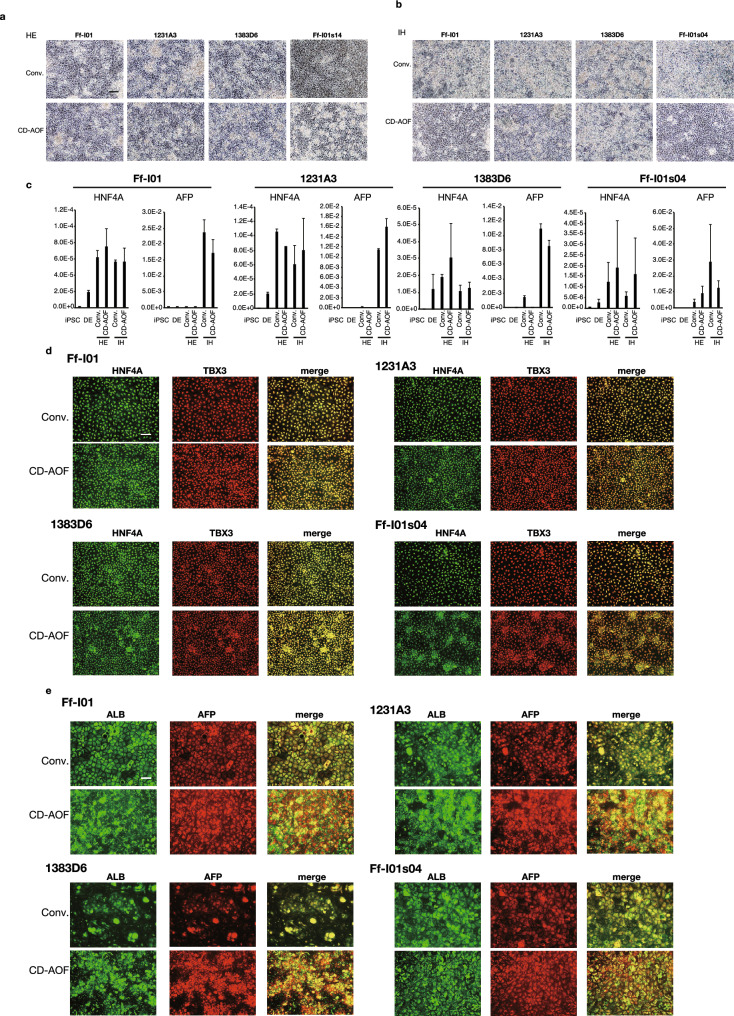
Generation of hepatic endoderm and immature hepatocytes using defined medium (Step II). (**a**, **b**) Morphological representation of hepatic endoderm (HE) and immature hepatocytes (IHs) differentiated from definitive endoderm using the conventional or CD-AOF medium. Scale bar, 100 µm. (**c**) Gene expression analysis of the HE and IHs differentiated using the conventional or the CD-AOF medium. Error bars represent standard deviation. (**d**) Immunostaining of the HE for Hepatocyte Nuclear Factor 4 (HNF4A) and T-Box Transcription Factor 3 (TBX3). Scale bar, 100 µm. (**e**) Immunostaining of IHs for albumin and Alpha Fetoprotein (AFP). Scale bar, 100 µm.

Step II of the all iPSC-LB generation involves the differentiation of DE to HE and IHs (Fig. 1). The conventional medium used in this stage is the Knockout™ Dulbecco’s modified Eagle’s medium (DMEM) supplemented with the KnockOut™ serum replacement. In this study, we replaced the conventional medium with the CD-AOF medium StemFit Basic03 (Basic03). Both media were supplemented with dimethyl sulfoxide (DMSO), L-glutamine, 2-mercaptoethanol (2-ME), and nonessential amino acids (NEAAs). The cell morphology was similar between the cells differentiated using the CD-AOF medium and the conventional medium (Fig. 3a,b). The HE and IH marker expression levels examined via quantitative reverse transcription-polymerase chain reaction (qPCR) were comparable between the cells differentiated using the conventional medium and those differentiated using the CD-AOF medium (Fig. 3c). The immunohistochemical analyses confirmed the appropriate differentiation of cells in the CD-AOF medium (Fig. 3d,e). We next examined whether the cells differentiated to IHs using the two media had similar capability to differentiate to MHs. Surprisingly, the expression levels of several markers and albumin secretion were significantly higher in the cells differentiated to IHs using the CD-AOF medium than in those differentiated using the conventional medium, although the same medium was used after the IH stage in both cultures (Figure S1a). These results suggested that the Basic03 medium could replace the conventional medium in Step II.

**Figure 4:**
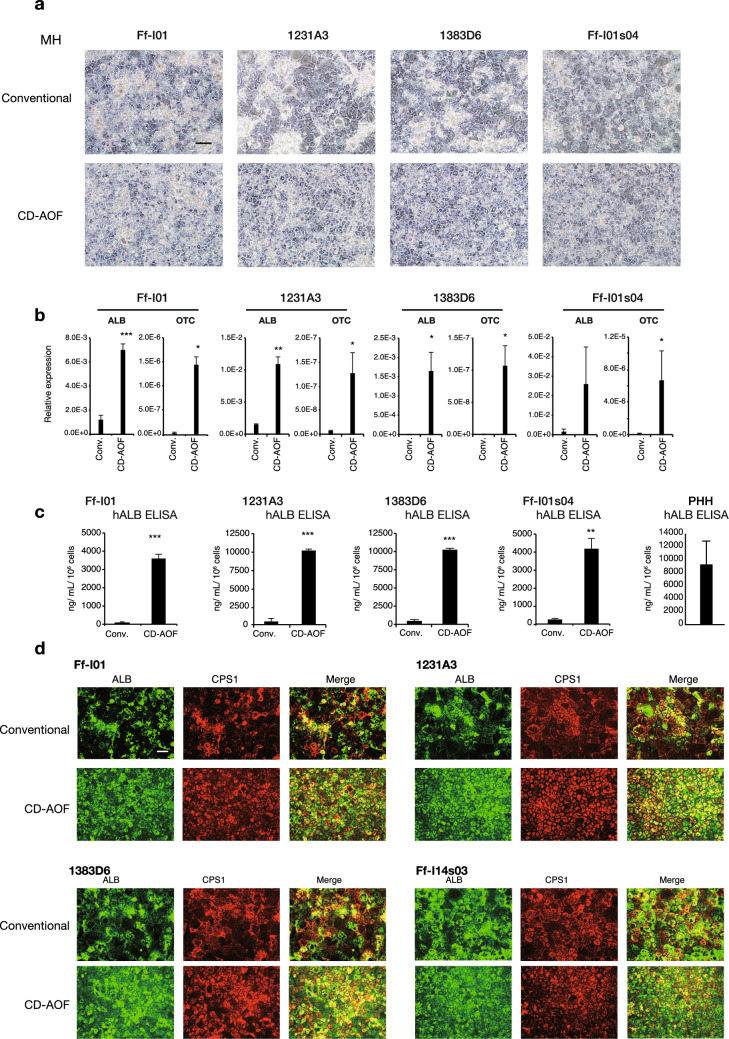
Generation of mature hepatocytes using defined medium (Step III). (**a**) Morphological representation of mature hepatocytes (MHs) differentiated from immature hepatocytes using the conventional or CD-AOF medium. Scale bar, 100 µm. (**b**) Gene expression analysis of the MHs differentiated using the conventional or the CD-AOF medium. Error bars represent standard deviation. Student’s *t* test, *p < 0.05, **p < 0.01, ***p < 0.001. (**c**) Albumin secretion at the MH stage in several iPSC lines. PHH: primary human hepatocytes. Data is presented as Student’s *t* test, *p < 0.05, **p < 0.01, ***p < 0.001. (**d**) Immunostaining of the MHs for albumin (ALB) and carbamoyl phosphate synthetase 1 (CPS1). Scale bar, 100 µm.

Step III of the all iPSC-LB generation involves the differentiation of IHs to MHs. We previously used the hepatocyte basal medium™ (HBM) containing the Single Quotes™ supplement except for Epidermal growth factor (EGF) (HCM™ without EGF) to differentiate IHs to MHs. We used DMEM instead of HBM and determined the necessity of Single Quotes™ supplement for differentiation. AS400 was used to replace serum, and dexamethasone and oncostatin M (OSM) were added in all conditions that were examined. Although the cellular morphology look similar, the MH produced using CD-AOF media (with or without Single Quotes™ supplement) have significantly better OTC and ALB expression and hALB secretion compared to conventional media (Fig. 4a–c, Fig. S1b, Fig. S1c). We assumed that the differences in gene expression profiles and albumin secretion observed between the two cell cultures using the two media resulted from the different media used in Step II. The immunohistochemical analyses confirmed the proper MH differentiation (Fig. 4d). Thus, these results indicated that HBM could be replaced with DMEM and that comparable findings were obtained irrespective of individual supplements.

We also evaluated the efficiency of the newly developed CD-AOF medium for other iPSC lines. All aforementioned experiments were performed for each cell type (Figs. 2, 3, 4), and gene expression levels were compared between the cells differentiated using the conventional and the CD-AOF media. Variable but insignificant differences in gene expression profiles were observed among the four iPSC lines differentiated in the conventional and the CD-AOF media, except for the higher gene expression levels and albumin secretion observed in the MHs that were differentiated using the CD-AOF medium in Step II (Figure S1a).

### AS400 as an alternative for B27 in differentiating ECs and STM/MCs

**Figure 5:**
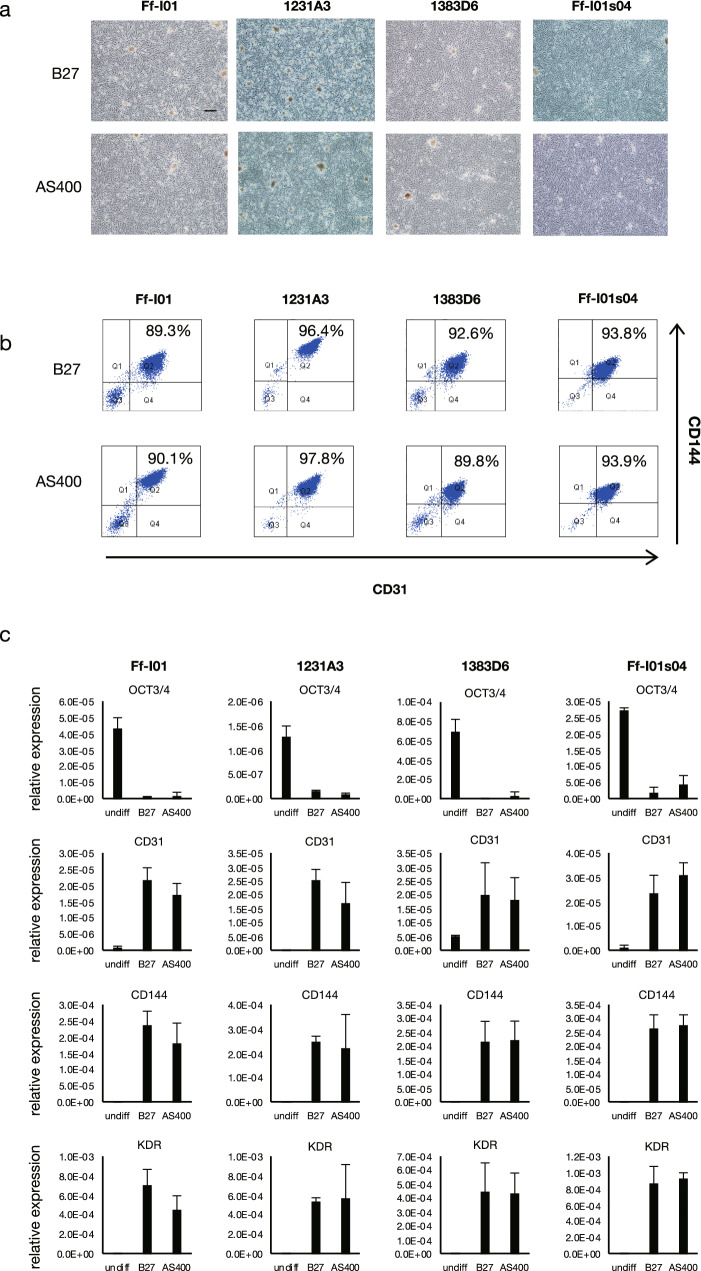
Generation of endothelial cells using defined medium (Step IV). (**a**) Morphological representation of endothelial cells (ECs) differentiated from iPSC using the conventional or CD-AOF medium. Scale bar, 200 µm. (**b**) Flow cytometric analysis of the ECs differentiated using the conventional or CD-AOF medium. (**c**) Gene expression analysis of the ECs differentiated using the conventional or CD-AOF medium. Error bars represent standard deviation.

**Figure 6:**
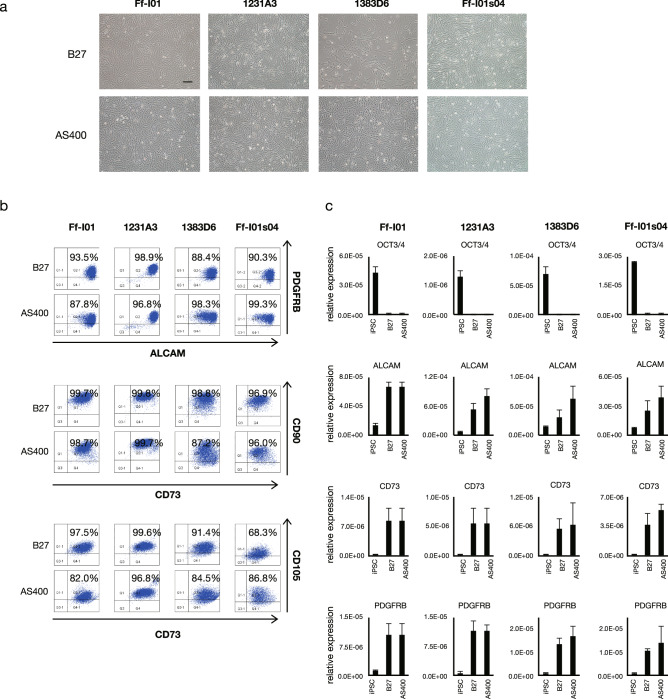
Generation of mesenchymal cells using defined medium (Step V). (**a**) Morphological representation of mesenchymal cells (MCs) differentiated from iPSCs using the conventional or the CD-AOF medium. Scale bar, 200 µm. (**b**) Flow cytometric analysis of the MCs differentiated using conventional or CD-AOF medium. (**c**) Gene expression analysis of the MCs differentiated using the conventional or CD-AOF medium. Error bars represent standard deviation.

As described above, AS400 was used successfully instead of B27 for DE derivation. We examined whether AS400 could replace B27 to generate ECs and STM/MCs. The cell morphology was similar between the cells differentiated using the media supplemented with AS400 and those differentiated using the media supplemented with B27 (Figs. 5a,6a). Therefore, AS400 could replace B27 during the induction of mesodermal lineages. The ECs derived from the mesoderm exhibited a CD31/CD144 double positivity rate of approximately 95% (Fig. 5b) and similar gene expression profiles (Fig. 5c). The STM/MCs derived from these lineages exhibited a Platelet-derived growth factor (PDGF) BB/ Activated Leukocyte Cell Adhesion Molecule (ALCAM) double positivity rate of approximately 85–99% (Fig. 6b) and similar gene expression profiles (Fig. 6c). These parameters were evaluated in several other iPSC lines, which confirmed that AS400 could also replace B27 for the differentiation of mesoderm-derived cell lineages. Taken together, these results indicated that mesodermal ECs and STM/MCs could be successfully generated from iPSCs using the CD-AOF medium.

### Generation of all iPSC-LBs from cells differentiated with the CD-AOF media

**Figure 7:**
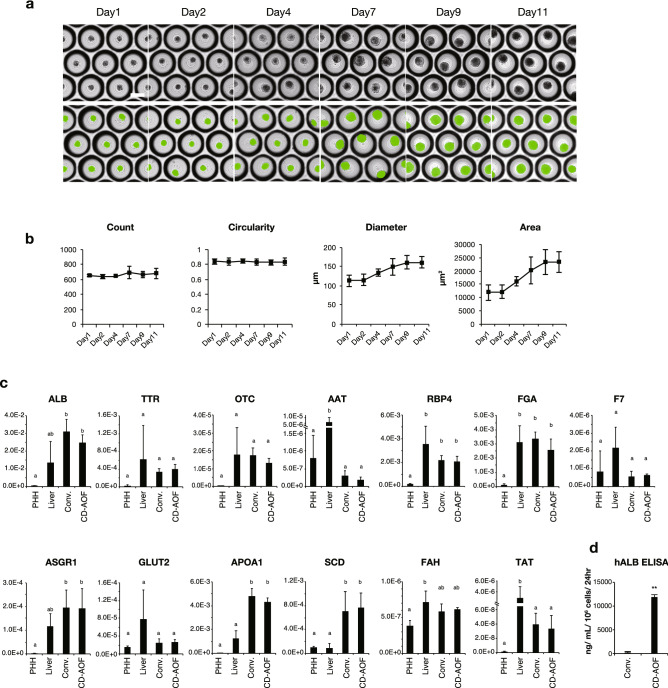
Generation of all iPSC-LBs using defined medium (Step VI). (**a**) Morphological representation of all iPSC-LBs generated using cells differentiated in the CD-AOF medium. Green segments in the bottom panel represent successful recognition of individual LBs in dimple-well plates. Scale bar, 200 µm. (**b**) Morphological analysis of the LBs generated using cells differentiated in the CD-AOF medium. (**c**) Gene expression analysis of the LBs generated using cells differentiated in the conventional or CD-AOF medium. Error bars represent standard deviation. Bars with the different letters are significantly different according to one-way ANOVA (analysis of variance) with post-hoc Tukey HSD (honestly significant difference) test (*p* < 0.05). See supplementary note for detailed interpretations. PHH: primary human hepatocytes. Liver: human liver tissue. (**d**) Human albumin secretion in iPSC-LBs differentiated in the conventional or CD-AOF medium.

Before generating LBs using cells differentiated from iPSCs, we confirmed that there were no residual undifferentiated iPSCs in cell populations differentiated using either the CD-AOF or the conventional medium (Table S1). Next, we used all developed media to generate all iPSC-LBs. The HE cells, ECs, and STM/MCs were mixed and seeded on Elplasia micro-dimple plates. We examined the appropriate timing for generating LBs from the HE. We examined the DEs 2, 4, 6, and 8 days after the DE stage (8, 10, 12, 14 days from the iPSC stage, respectively) and observed that day 4 or 6 after the DE stage was better than the other days (Fig. S2). We used cells on day 4 after the DE stage (day 10 from the iPSC stage) for further evaluation. The DMEM:KBM-VEC1 basal medium (1:1) supplemented with AS400 was used as the CD-AOF medium to culture LBs (step VI, see “Methods”). The resultant LBs were examined by whole-well imaging and quantification using a 3D cell scanner. The green segments in bottom panels of Fig. 7a represent the successful recognition of the LBs in micro-dimple plates. All iPSC-LBs generated using the CD-AOF medium exhibited high circularity and low variation in size (area, µm^2^) (Fig. 7b).

### Functional analysis of all iPSC-LBs generated with the CD-AOF medium

**Figure 8:**
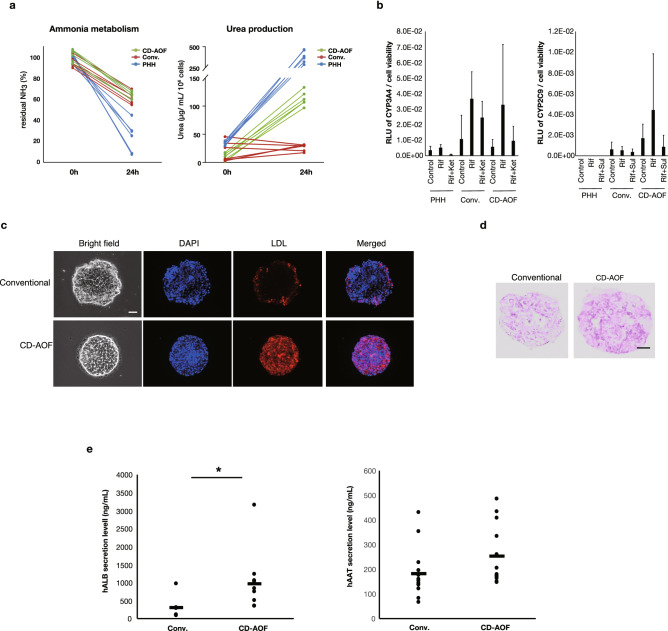
Functional analysis of the generated iPSC-LBs. (**a**) Ammonia metabolism and urea production in all iPSC-LBs differentiated with the CD-AOF media. (**b**) Cytochrome P450 (CYP) 3A4 and CYP2C9 induction and activity in all iPSC-LBs differentiated with the CD-AOF medium. (**c**) Lipid incorporation study in all iPSC-LBs by using DiI-Ac-LDL (Acetylated Low Density Lipoprotein labeled with 1,1'-dioctadecyl-3,3,3',3'- tetramethylindo-carbocyanine perchlorate). Scale bar, 50 µm. (**d**) Periodic acid-Schiff staining showing glucose incorporation in all iPSC-LBs. Scale bar, 20 µm. (**e**) All iPSC-LBs prepared with the CD-AOF or the conventional medium (see text for detail) were transplanted into the renal subcapsule of immunodeficient mice. Serum was collected to evaluate human serum albumin (HSA) and alpha-1 antitrypsin (AAT) concentrations two weeks after the transplantation. *p < 0.05.

The expression levels of mature hepatic marker genes were comparable between all iPSC-LBs generated using the CD-AOF medium and those generated using the conventional medium and CD-AOF samples show no significant inferior gene expression levels compared to liver tissue and primary human hepatocyte except for AAT and TAT (Fig. 7c). Albumin secretion was higher in all iPSC-LBs generated using the CD-AOF medium compared to those generated using the conventional medium (Fig. 7d). We next assessed ammonia metabolism to determine mature hepatic metabolic function. The clearance of ammonia from the culture medium and the secretion of urea were detected in the LBs cultured using the CD-AOF medium (Fig. 8a). The analyses of cytochrome P450 (CYP) activity (Fig. 8b), low density lipoprotein (LDL) incorporation (Fig. 8c), and glycogen storage (Fig. 8d) also indicated the functional maturation of all iPSC-LBs generated using the CD-AOF medium. We further tested the in vivo function of LBs generated using the CD-AOF medium. We used the Basic03 medium in step II of the conventional differentiation because the LB function was otherwise low. The mice transplanted with all iPSC-LBs generated using the CD-AOF medium in all steps exhibited higher serum human albumin secretion than those transplanted with all iPSC-LBs generated using the conventional medium (Fig. 8e). Alpha-1 antitrypsin (AAT) secretion was detected in the sera of both groups of mice. Overall, these data indicated that all iPSC-LBs generated using the CD-AOF medium displayed improved albumin secretion in vivo. Thus, the conventional medium was successfully replaced with the CD-AOF medium.

## Discussion

In the dawning era of regenerative medicine using iPSCs^3,17,18^, emerging approaches including organoid technology have accelerated and broadened the use of iPSCs in certain diseases^19–24^. However, several obstacles remain in translating iPSC technologies to clinical use, including the use of CD-AOF medium that meets the relevant safety and regulatory requirements. We have recently described the generation of multicellular liver organoids, iPSC-LBs, that provide functional rescue via transplantation in acute liver failure^11^. Furthermore, we have recently reported the mass production of all LB component cells, i.e., HEs, ECs, and MCs, from single-donor iPSCs, in all iPSC-LBs^13^. One drawback of all iPSC-LBs is the increasing complexity and the necessity of preparing multiple cell types under clinically applicable conditions. In the present study, we described the development of the CD-AOF medium for every cell type that constitute all iPSC-LBs. Our first goal was replacing the components of the CD-AOF medium without reducing the LB function. Surprisingly, however, there were several aspects, including albumin secretion, in which our newly developed medium was superior to the conventional medium used in several studies conducted by different laboratories. We also confirmed the absence of residual undifferentiated iPSCs in the differentiated cell populations generated using the CD-AOF medium, which should eliminate the risk of teratoma formation after transplantation.

In the present study, we employed the coating matrix containing the laminin 511E8 fragment to differentiate hepatocytes, ECs, and MCs. The fragment was used to maintain undifferentiated iPSCs, and this matrix could be used without adaptation to another matrix following the induction of differentiation. B27, which contains animal-derived components, is widely used to differentiate and maintain stem cells and primary cells. We demonstrated that the defined supplement AS400 could replace B27 at least for the derivation of DE, mesoderm-derived cell lineage, ECs, and MCs. Furthermore, we assessed whether AS400 could replace B27 for ectoderm-derived cell lineage differentiation. We differentiated neural crest cells (NCCs) from the ectoderm using a previously reported protocol and found that AS400 supported ectodermal lineage cell differentiation (Fig. S3)^25,26^. These results lend further support for the suitability of AS400 in the derivation of LBs and suggest that AS400 could be used instead of B27 to generate cell therapy products from three germ layers using CD-AOF media.

In conclusion, we have developed a CD-AOF medium for deriving the constituent cell types of all iPSC-LBs and have established the protocols for generating all iPSC-LBs under clinically applicable conditions. This protocol should also be valuable in generating hepatic cells in 2D cultures for therapeutic and basic research applications.

## Methods

### Human iPSC cultures

The human iPSC lines Ff-I01, 1231A3, 1383D6, and Ff-I01s04 were provided by Kyoto University. All iPSC lines were maintained on dishes coated with the laminin 511E8 fragment (iMatrix-511™, kindly provided by Nippi) in StemFit AK03N (AK03N) medium (Ajinomoto). The use of human iPSCs was approved by the ethics committee of Yokohama City University.

### Differentiation of human iPSC-DE, iPSC-HE, iPSC-IHs, and iPSC-MHs using the CD-AOF medium

The medium for differentiating hepatocytes was modified from our original report as follows^27^. On day zero, the iPSCs maintained in AK03N were dissociated using Accutase and seeded on dishes with differentiation medium in the presence of the Rho kinase inhibitor Y-27632 (Fujifilm Wako Pure Chemical). The dishes were coated with iMatrix-511 (Nippi) prior to or simultaneously with cell seeding^6,7^.

For differentiating the iPSCs to the DE in step I, the RPMI 1640 medium supplemented with 20% AS400 and 100 ng/ml Activin A (Ajinomoto) was used for six days. CHIR99021 (2 µM; Cayman Chemical) was added to the medium for the first three days, and sodium butylate (0.5 mM; Sigma Aldrich) was added to the medium from day 1 to day 4. The medium was changed every day. Human recombinant Activin A protein was manufactured by Ajinomoto, and its eligibility was approved by the Pharmaceuticals and Medical Devices Agency in Japan. AS400 was modified from AK03N, which was developed by Ajinomoto. All ingredients have been chemically defined, and none of the ingredients originate from animal sources. Large-scale manufacturing of AS400 has been achieved in well-validated and qualified factories in Japan. APC-conjugated mouse anti-human CD184 (555976, BD Pharmingen) was used for flow cytometric analysis.

For differentiating the HE and IHs from the DE in step II, the cells were incubated in the CD-AOF medium Basic03 supplemented with 1% DMSO (Nacalai Tesque), 0.1 mM 2-ME, 0.5% L-glutamine, and 1% NEAAs (Thermo Fisher Scientific) for seven days, and the medium was changed every day.

For differentiating the MHs from the IHs in step III, the cells were incubated in DMEM supplemented with 5% AS400, dexamethasone (Sigma Aldrich), and OSM (R&D Systems) for eight days, and the medium was changed every two days.

### Differentiation of human iPSC-DE, iPSC-HE, iPSC-IHs, and iPSC-MHs using the conventional method

The cells were differentiated as described previously. Briefly, the cells were incubated in RPMI 1640 (Thermo Fisher Scientific) supplemented with 2% B27, 50 ng/ml Wnt-3a, and 100 ng/ml activin A for six days to derive the DE. KnockOut DMEM medium (Thermo Fisher Scientific) supplemented with 20% KnockOut serum replacement (Thermo Fisher Scientific), 1% DMSO, 0.1 mM 2-ME, 0.5% L-glutamate, and 1% NEAAs was used to derive the HE and IHs. HBM (Lonza Bioscience) supplemented with the Single Quotes™ kit without EGF (HCM without EGF), 5% fetal bovine serum (FBS), dexamethasone, and OSM was used to derive MHs.

### Differentiation of human iPSC-ECs, STM, and NCC differentiation

ECs and MCs were derived as described previously^13^. iPSC-NCCs were derived using ectoderm-derived lineages, as described previously^25,26^. B27 was replaced with 20% AS400 to supplement the CD-AOF medium. Following antibodies were used for flow cytometric analysis: PE-conjugated mouse anti-human CD140b (PDGFRB; 558821, BD Biosciences); APC-conjugated anti-human CD166 (ALCAM; 130-119-809, Miltenyi Biotec); anti- human CD105, human CD73, and human CD90 (MSC phenotyping kit; 130-095-198, Miltenyi Biotec); FITC-conjugated mouse anti-human CD31 (557508, BD Biosciences); PE-conjugated mouse anti-human CD144 (561714, BD Biosciences).

### Generation of iPSC-LBs

The detailed procedures for differentiating hepatocytes were described previously^27^. Briefly, all cells were dissociated with TrypLE™, and the dissociated cells were incubated in the presence of Y-27632. The LBs were cultured with 50% HCM without EGF and 50% KBM-VEC1 basal medium (Kojin Bio), for the conventional medium 50% HCM without EGF supplemented with 5% FBS and 50% EGM was used. For the experiments using the CD-AOF medium, the LBs were cultured with 50% DMEM and 50% KBM-VEC1 basal medium supplemented with 2.5% AS400. Both media were supplemented with dexamethasone and OSM. Half of the medium was replaced daily. The LBs were evaluated using a Cell^3^iMager duos (SCREEN Holdings).

### Gene expression analysis by qPCR

qPCR analyses were performed following standard procedures with the RNeasy^®^ mini kit (Qiagen). qPCR was performed using the Universal ProbeLibrary System (Roche Molecular Systems). All probes and primers used for qPCR are presented in Table S2. The relative expression levels were normalized by the amount of 18S rRNA in each sample.

### Transplantation of the iPSC-LBs

The LBs were transplanted to the renal capsules of NOG (NOD.Cg-PrkdcscidIl2rgtm1Sug/ShiJic) mice (Central Institute for Experimental Animals). The mice were maintained according to the Yokohama City University institutional guidelines for the use of laboratory animals. All animal experiments were approved by the Institutional Animal Care and Use Committee of the Yokohama City University.

### Enzyme-linked immunosorbent assay

Albumin levels in medium were measured using enzyme-linked immunosorbent assay (ELISA). The medium samples were collected on day eight after LB generation, 24 h following the change of medium. The data was presented as ng/mL/10^6^ cells/24 h. The blood samples were collected 2 weeks after the transplantation of LBs into the renal capsule and were allowed to clot in centrifuge tubes for approximately five minutes at room temperature. All samples were centrifuged at 3000*g* for 10 min to collect serum. Human albumin levels in the medium and mouse serum samples were measured using the human albumin ELISA kit (Bethyl Laboratories). Human AAT levels in mouse serum samples were measured using the human AAT AssayMax ELISA kit (AssayPro), according to the manufacturer’s instructions. Adult human hepatocytes were purchased from manufacturers (dBioIVT, Kurabo).

### Measurement of ammonia and urea levels

To assess ammonia metabolism, the LBs were loaded with 2 mM NH_4_Cl on day seven after the formation of LBs, and the LBs were incubated for 24 h. The concentrations of NH_4_Cl remaining in the medium were measured by the ammonia test kit II (Arkray), according to the manufacturer’s instructions. To assess urea production, the medium was changed on day seven after the formation of LBs, and the LBs were incubated for 24 h prior to analysis. The medium was collected and measured by the QuantiChrom™ urea assay kit (BioAssay Systems), according to the manufacturer’s instructions.

### CYP assay

CYP measurement was performed on day eight following the formation of LBs using the P450-Glo™ assay (Promega), according to the manufacturer’s instructions. Rifampicin with or without ketoconazole or sulfaphenazole was loaded eight hours before the collection of medium for analysis.

### Periodic acid-Schiff stain

Frozen LB sections prepared eight days after their formation were stained with Schiff’s reagent according to a standard protocol.

### Immunostaining

Cells were fixed with 4% paraformaldehyde for 15 min and washed twice with phosphate-buffered saline. The cell membrane was permeabilized using 0.1% Triton X-100 in phosphate-buffered saline for 10 min. After blocking the cells with 5% FBS in phosphate-buffered saline, the cells were incubated with one or two of the following primary antibodies followed by a secondary antibody: FOXA2 (07-633, Millipore), SOX17 (AF1924, R&D Systems), HNF4A (sc-6556, Santa Cruz Biotechnology), TBX3 (ab99302, Abcam), CPS1 (Hepatocytes Monoclonal Antibody (OCH1E5); MA5-12417, Thermo Fisher Scientific), AFP (A8462, Sigma), and albumin (A6684, Sigma).

### Statistical analyses

Data were expressed as means ± standard deviation of independent experiments. All experiments were performed at least three different cell preparations. Statistical significance was assessed by Student's *t* test for gene expression analyses, and statistical significance was assessed by the nonparametric Mann–Whitney *U* test for the albumin amount secreted in cultures. Two-tailed *p* values of < 0.05 were considered statistically significant. One-way ANOVA (analysis of variance) with post-hoc Tukey HSD (honestly significant difference) test was employed for analysing data presented in Fig. 7.

## Supplementary information

Supplementary Information.
