# Supplementary material for: Generation of human induced pluripotent stem cell-derived liver buds with chemically defined and animal origin-free media
Source: Sci Rep. 2020 Oct 21;10:17937. doi: 10.1038/s41598-020-73908-1 (PMC7578079; doi:10.1038/s41598-020-73908-1)
Supplement: Supplementary file 1 — Supplementary Information. [file 41598_2020_73908_MOESM1_ESM.pdf]

Generation of human induced pluripotent stem cell-derived liver buds with chemically defined and animal origin-free media

Keisuke Sekine, Shimpei Ogawa, Syusaku Tsuzuki, Tatsuya Kobayash, Kazuki Ikeda, Noriko Nakanishi, Kenta Takeuchi, Eriko Kanai, Yugo Otake, Satoshi Okamoto, Tsuyoshi Kobayshi, Takanori Takebe, and Hideki Taniguchi

Supplementary Note

Detailed clarification of interpretation associated to Fig 7c;

ALB: PHH differ from Conv and CD-AOF while not differ from Liver. Liver do not differ from any other sample.

TTR: No significant difference among all samples.

OTC: No significant difference among all samples.

AAT: Liver differ from other 3 samples.

RBP4: PHH differ from other 3 samples.

FGA: PHH differ from other 3 samples.

F7: No significant difference among all samples.

ASGR1: PHH differ from Conv and CD-AOF while not differ from Liver. Liver do not differ from any other sample.

GLUT2: No significant difference among all samples.

APOA1: PHH and Liver differ from Conv and CD-AOF. PHH vs Liver or Conv vs CD-AOF show no significant difference.

SCD: PHH and Liver differ from Conv and CD-AOF. PHH vs Liver or Conv vs CD-AOF show no significant difference.

FAH: PHH differ from Liver.

TAT: Liver differ from other 3 samples.

Supplementary Figure Legends

Figure S1. Hepatic and liver bud function using defined medium. (a) immature hepatocytes (IHs) derived with the chemically defined, animal origin-free (CD-AOF) or the conventional medium were evaluated at the MH stage in several iPSC lines. HCM (without EGF) was used to differentiate IHs to MHs in both conditions. IHs derived using the CD-AOF medium outperformed when evaluated at the MH stage even same media were used to differentiate the IHs to MHs. (b, c) Evaluation of supplements in Single Quotes kit in mature hepatocytes (MHs). The gene expression (c), and albumin secretion (c) were comparable between the MHs differentiated in Dulbecco's modified Eagle medium without any supplements and those differentiated in DMEM with supplements.

Figure S2. Examination of the timing to generate LBs after the DE stage. We examined the HE timing to generate LBs after DE. Four days after the DE stage (10 days from the iPSC stage) was selected for further evaluation.

Figure S3. Generation of neural crest cells using defined medium. (a) Morphological representation of neural crest cells differentiated using medium supplemented with AS400 or B27. Scale bar, 200 µm. (b) Flow cytometric analysis of CD271 expression showing no significant difference between the two conditions.

Figure S1

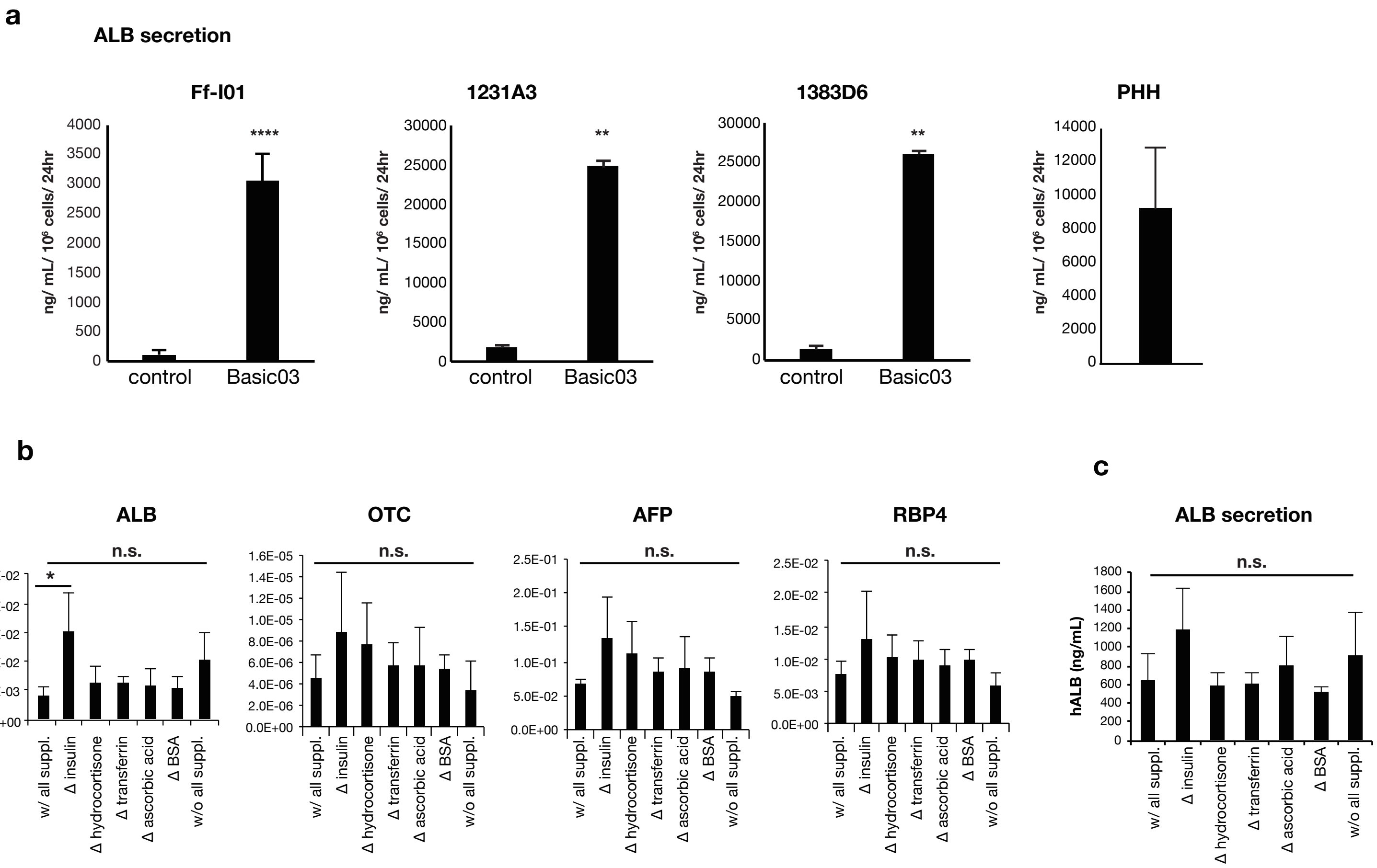
a
ALB secretion
Ff-I01
1231A3
1383D6
PHH
ng/ mL/ 10⁶ cells/ 24hr
****
**
**
control
Basic03
b
ALB
OTC
AFP
RBP4
n.s.
*
w/ all suppl.
Δ insulin
Δ hydrocortisone
Δ transferrin
Δ ascorbic acid
Δ BSA
w/o all suppl.
c
ALB secretion
hALB (ng/mL)
n.s.

Figure S2

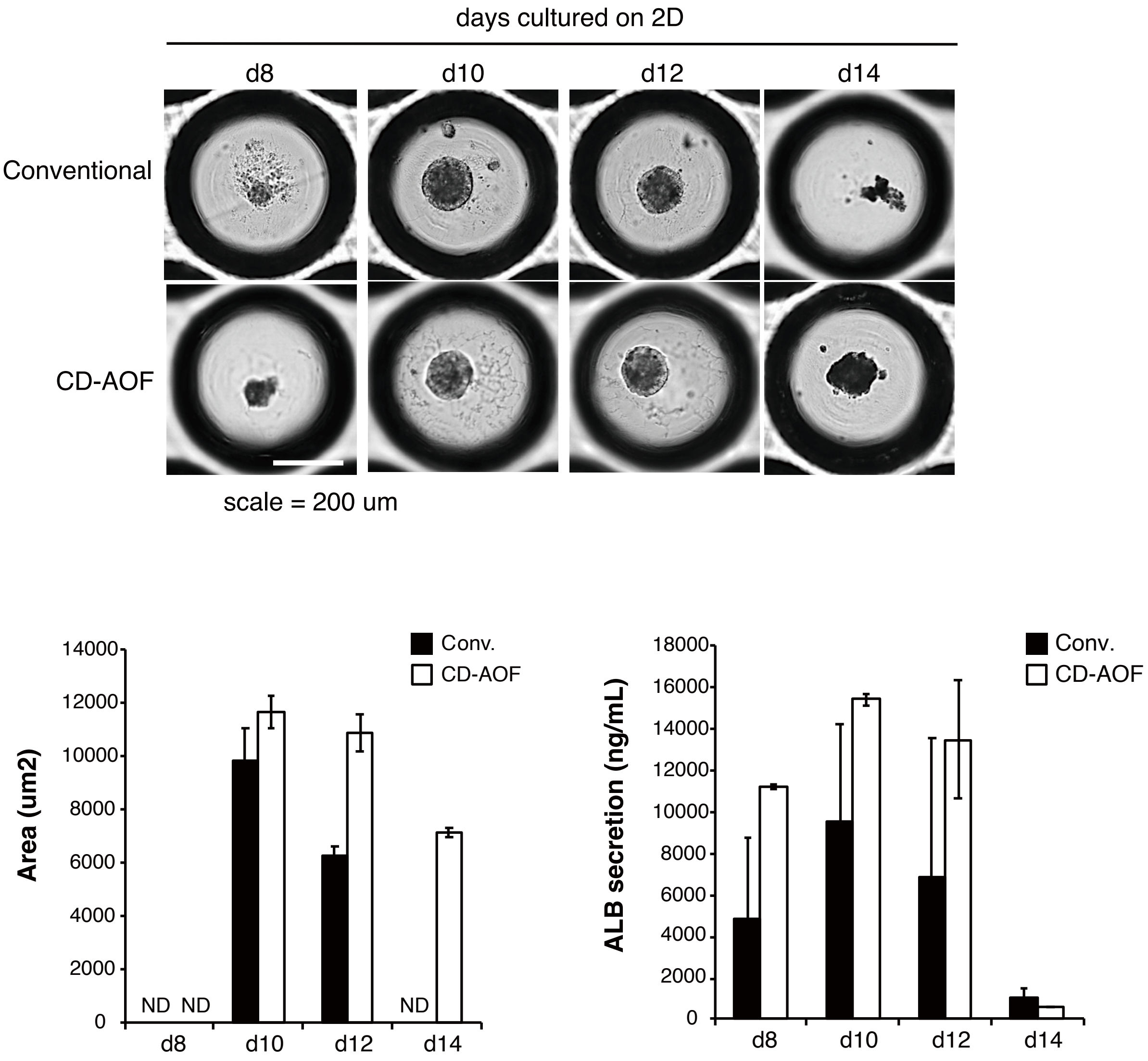
days cultured on 2D
d8
d10
d12
d14
Conventional
CD-AOF
scale = 200 um
Area (um2)
14000
12000
10000
8000
6000
4000
2000
0
ND
ND
ND
d8
d10
d12
d14
Conv.
CD-AOF
ALB secretion (ng/mL)
18000
16000
14000
12000
10000
8000
6000
4000
2000
0
d8
d10
d12
d14
Conv.
CD-AOF

Figure S3

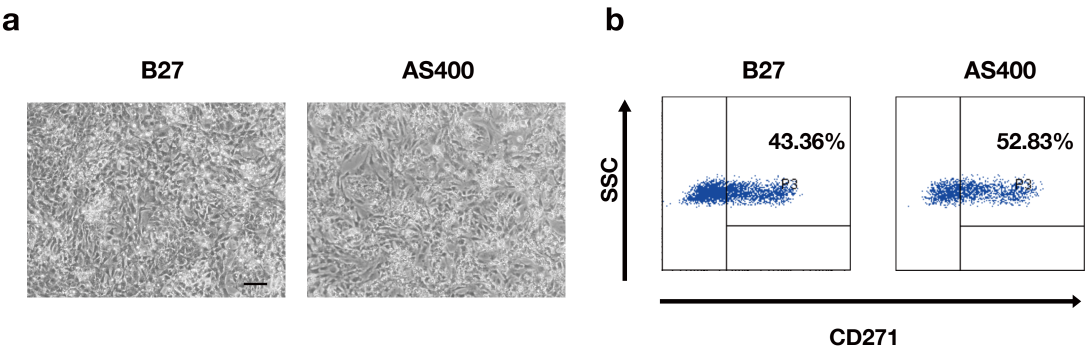

Table S1. Number of residual undifferentiated induced pluripotent stem cells in differentiated cell populations

	Colony number (colonies /cm^2)	N
Conventional Media	0	31
CD-AOF media	0	12

Table S2. Primers and probes used for qPCR in this study

	Forward	Reverse	Probe No.
POU5F1 (OCT3/4)	CTTCGCAAGCCCTCATTTC	GAGAAGGCGAAATCCGAAG	60
CXCR4	ATTGGGATCAGCATCGACTC	CAAACTCACACCCTTGCTTG	79
SOX17	ACGCCGAGTTGAGCAAGA	TCTGCCTCCTCCACGAAG	61
HNF4A	TCAGACCCTGAGCCACCT	AGCAACGGACAGATGTGTGA	27
AFP	TCCTTGTAAGTGGCTTCTTGAAC	TGTACTGCAGAGATAAGTTTAGCTGAC	61
ALB	AATGTTGCCAAGCTGCTGA	CTTCCCTTCATCCCGAAGTT	27
OTC	CACCTTCAGGCAGCTACTCC	TGGTACCATTCTCTTTGGCATA	53
RBP4	CCAGAAGCGCAGAAGATTG	TTTCTTTCTGATCTGCCATCG	17
ALCAM	CAGTTCCTGCCGTCTGCT	CTGAATTTACAGTATACCATCCAAGG	34
CD73	GCACTATCTGGTTCACCGTGT	CCTTCCACACCATTATCAAATTC	48
PDGFRB	CATCTGCAAAACCACCATTG	GAGACGTTGATGGATGACACC	10
PECAM1 (CD31)	CAGAGAGACCGGCTGTGG	CATTGTTCCCGGTTTCCA	46
CDH5 (CD144)	CATGAGCCTCTGCATCTTCC	GCAGTCCAACGGAACAGAA	30
KDR	GAACATTTGGGAAATCTCTTGC	CGGAAGAACAATGTAGTCTTTGC	18
APOA1	CCTTGGGAAAACAGCTAAACC	CCAGAACTCCTGGGTCACA	39
SCD	CCTAGAAGCTGAGAAACTGGTGA	ACATCATCAGCAAGCCAGGT	82
F7	CCTTCTGCTTGGGCTTCA	GGCATGTCCCGTGTTTCT	30
ASGR1	TGACCACCATCAGCTCAGAA	ACAGACAACCACAAGCAGCA	8
